# Uric acid promotes myocardial infarction injury via activating pyrin domain-containing 3 inflammasome and reactive oxygen species/transient receptor potential melastatin 2/Ca^2+^pathway

**DOI:** 10.1186/s12872-023-03040-1

**Published:** 2023-01-10

**Authors:** Haiyun Wu, Ruozhu Dai, Min Wang, Chengbo Chen

**Affiliations:** Department of Cardiology, Quanzhou First Hospital Affiliated to Fujian Medical University, No. 250 East Street, Quanzhou, 362000 China

**Keywords:** Uric acid, Cardiomyocytes injury, NLRP3 inflammasome, Inflammatory factors, TRPM2 channel

## Abstract

Cardiomyocytes injury has been considered as a key contributor for myocardial infarction (MI). Uric acid (UA) can induce cardiomyocytes injury, which is closely related to NLRP3 activation and inflammatory factor generation. However, the mechanism how UA modulates cardiomyocytes remains elusive. Western blotting and qRT-PCR were applied for measuring protein and mRNA expression, respectively. ROS production and Ca^2+^ influx were measured by flow cytometry. Patch clamp technique was used for measuring transient receptor potential melastatin 2 (TRPM2) channel. Ligation of left anterior descending for 2 h was performed to induce MI animal model. The rats were treated by different concentration of uric acid. The artery tissues were stained by HE and collected for measurement of NLRP3 and inflammatory factors. Supplementation of UA significantly promoted apoptosis, and augmented the expression of intercellular adhesion molecule-1, chemoattractant protein-1, vascular cell adhesion molecule-1, and NLRP3 inflammasome. Knockdown of NLRP3 reversed the influence of UA on MI by decreasing collagen deposition, fibrotic area, apoptosis. The expression of NLRP3 inflammasome increased markedly after treatment of UA. UA activated ROS/TRPM2/Ca^2+^ pathway through targeting NLRP3. UA activated NLRP3 inflammasome and augments inflammatory factor production, which in turn exacerbates cardiomyocytes injury. Knockdown of NLRP3 reversed the influence of UA on apoptosis and cell cycle. UA may promote cardiomyocytes injury through activating NLRP3 inflammasome and ROS/TRPM2 channel/Ca^2+^ pathway.

**Supplementary Information:**

The online version contains supplementary material available at 10.1186/s12872-023-03040-1.

## Introduction

Cardiovascular disease is a major public health problem affecting human health. Myocardial infarction (MI) is one of the main causes of sudden death. Although the method of rapid recovery of coronary blood flow is commonly used in clinic, which can restore the blood supply to the myocardium and improve the survival rate of patients with myocardial infarction. However, this method is not enough to prevent the further deterioration of cardiac function and might further cause serious problems such as ischemia–reperfusion injury. It has been reported that high uric acid (UA) is closely related with cardiovascular disease, and the severity of cardiac lesions is positively related to the level of serum UA [1, 2]. Meanwhile, UA can induce the endothelial dysfunction, and this effect might associate with decreased intracellular ATP, mitochondrial alterations [3, 4]. In addition, UA was reported to be associated with acute heart failure presentation in acute coronary syndrome patients [5]. Meanwhile, high admission levels of serum uric acid are independently associated with in-hospital adverse outcomes and mortality in a contemporary population of acute coronary syndrome patients [6].

Pyrin domain-containing 3 (NLRP3) inflammasome is a multiprotein complex comprising NLRP3 receptor protein, protease caspase-1 and apoptosis­associated speck­like protein (ASC) [7, 8]. NLRP3 is an intracellular sensor that measure environmental irritants, endogenous danger signals, microbial motifs, resulting in the formation and activation of the NLRP3 inflammasome [9]. NLRP3 inflammasome plays a key role in inflammatory responses, which is closely associated with lipid formation in vascular, a characterized event for atherosclerosis [10, 11]. Some other inflammatory factors such as monocyte chemoattractant protein-1 (MCP-1), intercellular adhesion molecule-1 (ICAM-1), and vascular cell adhesion molecule-1 (VCAM-1) play crucial role in recruitment of inflammatory monocytes into vascular wall and initiate atherosclerosis [12, 13]. Meanwhile, genetic deficiencies of MCP-1, VCAM-1, and ICAM-1 have been believed to be related with decreased atherosclerosis [14]. However, whether UA could promote the endothelial dysfunction by activating NLRP3 inflammasome and increasing inflammatory factors remains elusive.

Oxidative stress has been believed as a key contributor for the atherosclerosis, and the reactive oxygen species (ROS) induced by oxidized low-density lipoprotein could initiate endothelial dysfunction and further accelerate atherosclerosis [15]. transient receptor potential melastatin 2 (TRPM2) channel is responsible for the Ca^2+^ influx, and also known as a sensor for oxidative stress and ROS [16]. ROS induced Ca^2+^ influx through TRPM2 channel has been considered as an important regulator for inflammatory factors [17]. However, if UA induces endothelia dysfunction through targeting ROS/TRPM2 channel/ Ca^2+^ remains unclear.

In the present study, we investigated the influence of UA on the cardiomyocytes apoptosis. The effects of UA on the expression of MCP-1, VCAM-1, ICAM-1, and NLRP3 inflammasome were measured. Additionally, we conducted animal experiments to observe the morphological changes of myocardial tissue after treatment with different concentrations of UA. Finally, we proved that UA promote cardiomyocytes injury via upregulating NLRP3 inflammasome and activating ROS/TRPM2/Ca^2+^.This study may provide a new insight about the intervention and prevention of cardiomyocytes injury and MI.

## Methods

### Cell culture

Human cardiomyocytes (#SCC109, AC16, Merck, US) and human aortic endothelial cells (HAEC, #PCS-100-011, ATCC) were incubated with DMEM (#10,567,022, Thermo, US) containing 10% FBS (#10,099, Thermo, US), 90 g/mL heparin, and 1% penicillin/streptomycin at 37℃ and with CO_2_. After reaching 80% confluence, the cells were passaged with 0.3% trypsin–EDTA and seeded onto plats. After different treatment describe below, the cells were applied for different experiments. The cardiomyocytes used in this study were identified with cardiac markers (connexin 43 and SIRP-***a***), and presented in Additional file 1: Fig. S1.

### Measurement of apoptosis and cell cycle by flow cytometry

These experiments were performed as described previously [18, 19]. Firstly, the cells were treated either by different concentrations of UA (0, 4, 8, 12 mg/dL, respectively) for 12 h or by different incubation time (0, 6, 12, 24 h, respectively) with 8 mg/dL UA. UA was purchased from Sigma (#TR24321, US). Then, flow cytometry was used to measure cell apoptosis with Annexin V‑fuoresecin isothiocyanate (FITC) apoptosis measurement kit (#556,547 BD Biosciences, United State). Cells were collected and washed twice by cold PBS (#C0221A, Beyotime, China). 10^6^ cells were suspended in 200 µL binding buffer containing 5 µL propidium iodide and 10 µL Annexin V-FITC. Incubate cells in the dark for 30 min, and flow cytometry analysis (Agilent, Novocyte cytometer, USA) was conducted.

The cell cycle was performed as described below. Cells were treated with UA or siRNA for 24 h. The cells were digested with trypsin (0.2%). After fixation with 70% cold ethanol, the cells were incubated with RNase A for 20 min. Then, the cells were incubated with propidium iodide for 30 min in the dark. Cell cycle was analyzed with flow cytometry (Agilent, Novocyte cytometer, USA). Flowjo™ software v11 was used for flow cytometry data analysis. The gating strategy was set as follows. The cells without staining by PI and Annexin V-FITC were used for flow cytometry analysis. The gate was set to cover negative cells in the lower left quadrant. Then, the set gate was used to analyze apoptotic cells and dead cells in the lower right quadrant and higher right quadrant, respectively. FSC-H versus FSC-A strategy was used to discriminate doublets. The experiments were repeated at least 3 independent times. 3 samples were set in each group.

### Assessment of cell proliferation

The cell proliferation was measured as described previously [19]. The proliferation of cells was measured by MTT assay. Briefly, MTT reagent (#ST316, Beyotime, China) was added to the cells. After 3 h incubation the supernatant was removed and 200 µL DMSO (#ST038, Beyotime, China) was added. The optical density of each well at 450 nm was detected after 2 h incubation. The experiments were repeated at least 3 independent times. 3 samples were set in each group.

### Western blotting

The western blotting was performed as described previously [20]. The primary antibodies used in the present study were obtained from Abcam. Lysis buffer was used to prepare protein of cells. Same amounts of protein were loaded on an SDS-PAGE and then transferred electrophoretically to PVDF membrane (Millipore, USA). After blocking with TBST containing 5% milk, the membranes were incubated with primary antibody (1:1000) at 4 °C overnight. After washing and incubation, the membranes were incubated with secondary antibody (1:2000) in TBST. ECL Plus detection system (Millipore, USA) was applied for immuno-detection, e-Blot software was used for acquisition, and Image J software was used for analysis. The antibodies used in this study are listed as follows: rabbit monoclonal to NLRP3 (#ab263899, abcam, UK), rabbit polyclonal to ASC1 (#ab70627, abcam, UK), Rabbit monoclonal to caspase-1 (#ab207802, abcam, UK), Rabbit monoclonal to SIRP alpha (#ab302974, abcam, UK), Rabbit monoclonal to Connexin 43 (#ab214274, abcam, UK). The experiments were repeated at least 3 independent times. The original blots/gels are presented in Additional files 2, 3, 4: Figs. S2–S4, and the gels were cut prior to hybridisation with antibodies to save antibody, which is common in most labs. Therefore, we are unable to provided full-length blots/gels.

### RNA isolation and real-time PCR

The experiment was conducted following the method of previous study [21]. Briefly, Total RNA was extracted through TRIzol reagent (#15,596,026, Thermo, USA) and 500 ng RNA was reverse-transcribed into cDNA using the Primer Script RT reagent kit (#RR014B, Takara Bio, China). Real-time PCR was performed using SYBR Premix Ex TaqTM II kit (#RR390A, Takara Bio, China). The primers used for NLRP3, Caspase-1, and ASC were listed as follows: (1) NLRP3: forward: 5′-ATTACCCGCCCGACAATAGG-3′ and reverse: 5′-CATGAGTCAGCTAGGCTAGAA-3′; (2) Caspase-1: forward: 5′-TGGAAGGTAGGCAAGACT-3′ and reverse: 5′-ATAGTGGGCATCTGGGTC-3′; (3) ASC: forward: 5′- AACCCAAGCAAGATGCGGAAG-3′ and reverse: 5′- TTAGGGCCTGGAGGAGCAAG-3′. Each sample was replicated three times with no RT and no template control included. Data were analyzed by comparing cycle threshold values. The experiments were repeated at least 3 independent times.

### Measurement of MCP-1, VCAM-1, and ICAM-1 in the cell supernatant

The supernatants were collected and centrifuged to remove cell debris. MCP-1 (#PC128, Beyotime, China), VCAM-1 (#PV951, Beyotime, China), and ICAM-1 (#PI495, Beyotime, China) in the supernatant were detected with relative ELISA kits. The experiments were repeated at least 3 independent times. 3 samples were set in each group.

### Myocardial ischemia–reperfusion injury (MIRI) animal model

All the experimental protocols were approved by Independent Ethical Committee of Quanzhou First Hospital Affiliated to Fujian Medical University. Wistar rats (8 weeks, 200–220 g, Charles river, Beijing, China) were anaesthetized by intraperitoneal injection with ketamine (120 mg/kg) as describe previously [22]. The rats were housed in a standard cage under a 12 h light/dark cycle, and free available to food and water. Ligation of left anterior descending for 2 h was performed to induce MIRI. The ischemia of the anterior wall of left ventricular was confirmed by the ST-segment elevation on the electrocardiogram [23]. The average phenotype/infarct size of MIRI animal had been confirmed with our preliminary experiment. The animals in the group MIRI + UA were treated with UA (400 mg/kg) through gavage twice one day for 4 weeks. The rats in the group MIRI + UA + si-NLRP3 were treated with UA (400 mg/kg) and si-NLRP3 (100 µM, intravenous injection) twice one day for 4 weeks. The rats in the group sham and MIRI were treated with same amount of saline. The animal numbers were 8 for each group. The doses of UA and Si-NLRp-3 were decided based on our preliminary experiment and published references [24–26]. The animal numbers were 8 for each group with 4 male rats and 4 female rats.

### HE and Masson staining

Animals were euthanized at 4 weeks after surgery with prolonged exposure to isoflurane as described previously [27]. Tissues were fixed in formalin. The tissues were cut into 10 µm-thick sections. Sections were stained with HE and Masson methods, and visualized using Zeiss AxioVision 4.8 software. Serial sections were collected starting from disappearance of the ligation suture, and mounted on poly-D-lysinecoated slides. Five evenly spaced slides were chosen for HE and Masson staining. Measurements made from 5 separate slides at same position from ligation point were averaged for each group and this average was used for statistical analysis. The infarct area was quantified with image J software.

### Measurement of ROS and Ca2+ by flow cytometry

Cells were labeled in the dark with 2 mM DCFHDA–AM (#C6827, Invitrogen-Molecular Probes, USA) for 20 min firstly. After washing and re-suspending in PBS (#C0221A, Beyotime, China), the cells were analyzed by flow cytometer (Becton–Dickinson, C6 cytometer, USA) at an excitation wavelength of 514 nm. The Ca^2+^ in cell was measured in the similar way as described above except that cells were labeled with 10 μM Fluo-3AM (#F1241, Thermo, US) for 1 h. After washing with buffer free of calcium ions, cells were measured by flow cytometer (Agilent, Novocyte cytometer, USA) at an excitation wavelength of 480 nm. The experiments were repeated at least 3 independent times. 3 samples were set in each group.

### TRPM2 channel current measurement

The TRPM2 channel current measurement was performed as described previously [28]. EPC-10 amplifier (HEKA, Lambrecht/Pfalz, Germany) was use to record whole cell channel currents. Capillary glass tube (Sutter Instruments, USA) was pulled into electrodes with a micropipette puller. The electrodes were applied to contact cells using motorized micromanipulator (Sutter Instruments, USA) under an inverted microscope. The electrodes were sucked with negative pressure pump to obtain GΩ seal. After the GΩ seal was achieved, fast capacitor compensation was used and negative pressure was applied to break cell membrane for recording. Slow capacitor compensation was used and the membrane resistance and capacitance were recorded. As a control, TRPM2 was activated by ADP-Ribose (0.5 mM) in intracellular fluid after the membranes were broken. When TRPM2 channel current was stable, 2-APB was added for 5 min (or till the current was stable) and the current was recorded. Several individual cells were measured repeatedly and independently. The experiments were repeated at least 3 independent times.

### MI model validation

Transthoracic echocardiography examination was performed uing a Xario ultrasound device (Toshiba, Japan) with a 12 MHz cardiac probe after animal anesthesia. MP100-CE (BIOPAC Systems, USA) was applied for recording. The experiments were repeated at least 3 independent times. The model establishment was confirmed by the occurrence of ST-segment elevation in electrocardiogram. The ST-segment elevation electrocardiogram was presented in the Additional file 5: Fig. S5.

### Tunel staining

The sections were incubated with proteinase K (10 μg/mL, 30 min), hydrogen peroxide (0.4%, 25 min), and Triton X-100 (0.1%, 20 min), and washed with PBS (5 min). 5% goat serum (#ab7481, abcam, UK) was used for blocking, and BCIP/NBT liquid (#C1088, Beyotime, China) was used for incubating Sects. (2 h). The sections were mounted and observed with a fluorescence microscope (Zeiss, Germany). At least 5 random images in each group were used for apoptosis analysis.

### Statistical analysis

Assays were performed at least in three independent experiments with one representative picture presented. Data were presented as mean ± standard deviation (SD). Statistical analysis was performed using Graphpad Prism 4.0 (GraphPad, USA). One-way analysis of variance (ANOVA) was applied for Statistical evaluation among the different groups. *P* values < 0.05 was considered statistically significant.

## Results

### UA promoted cell death and impairs the proliferative activity of cells

**Fig. 1:**
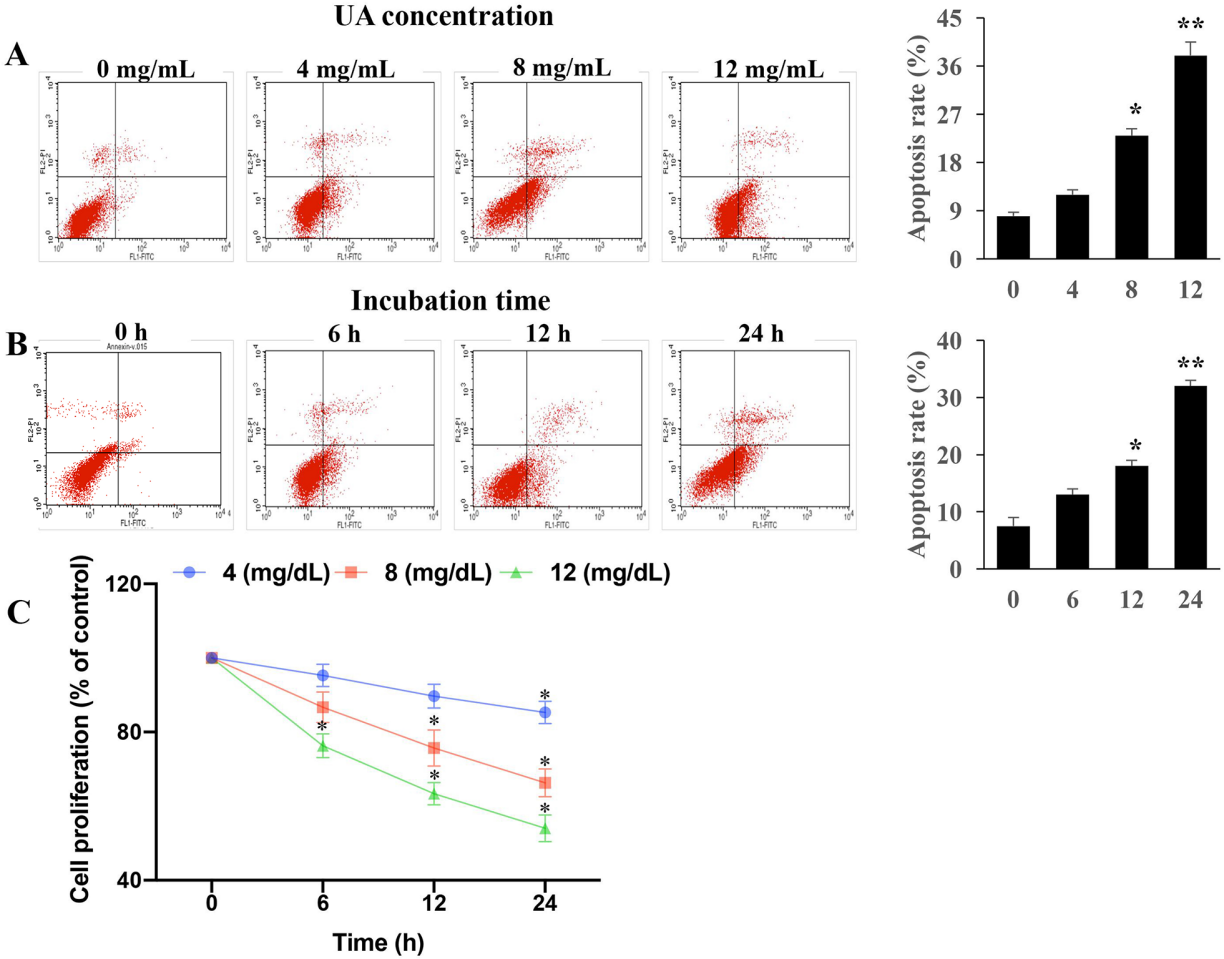
UA promoted apoptosis and inhibited cell proliferation. **A** UA enhanced the apoptosis of cells through dose-dependent manner; **B** UA promoted the apoptosis of cells through time-dependent manner; **C** UA suppressed the proliferation of cells. *P < 0.05 and **P < 0.01 compared with group 0 mg/dL UA or group 0 h incubation

As shown in Fig. 1A, B, supplementation of UA induced cell death in a dose- and time-dependent manner. Reciprocally, compared with the effects of low dose of UA, high dose of UA remarkably restricted the proliferative activity of cells (Fig. 1C). Therefore, our data reveal that UA treatment influences cell survival and leads to cell dysfunction.

### UA promoted the expression of NLRP3, ASC and Caspase-1 in cells, and increased the secretion of inflammatory cytokines from cells

**Fig. 2:**
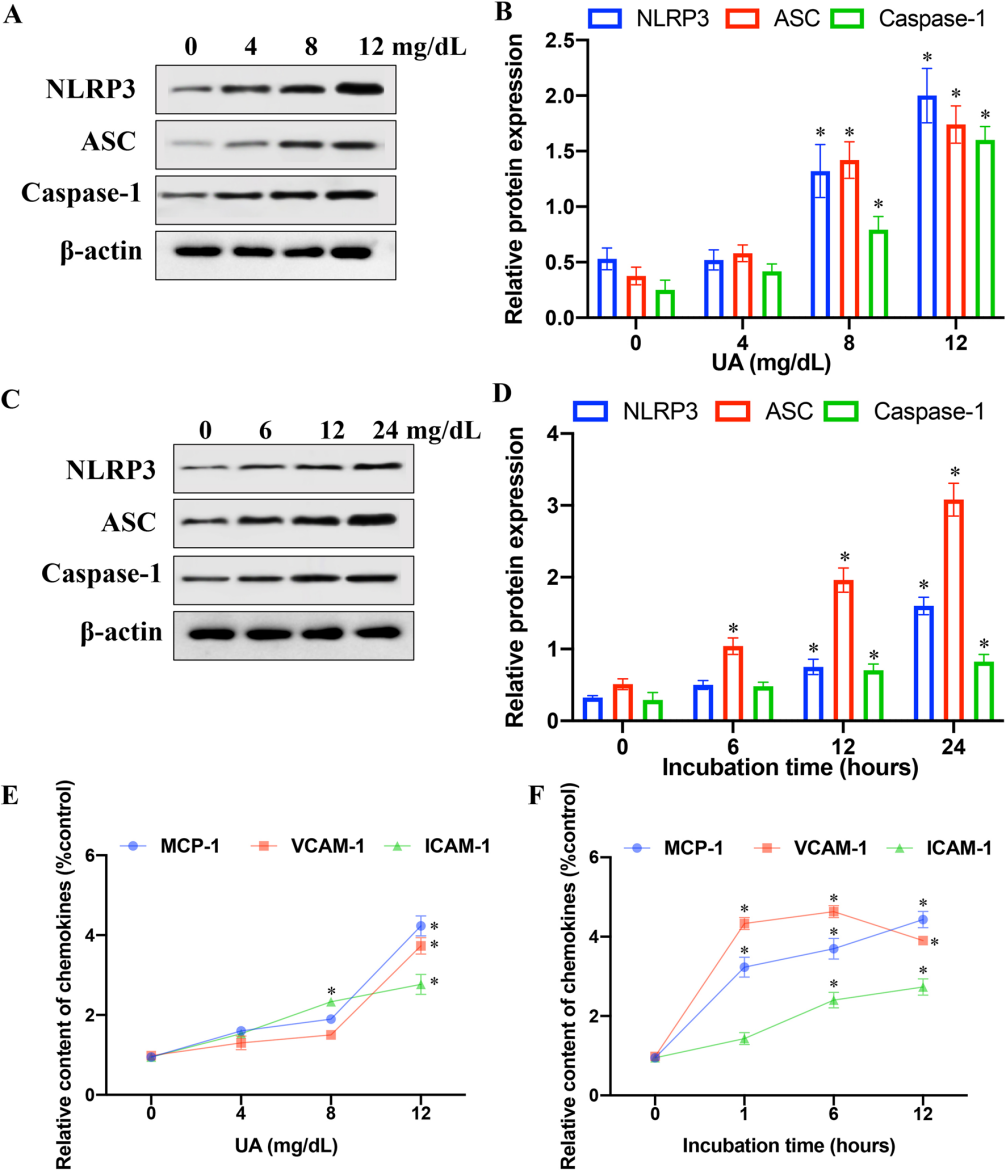
UA promoted the expression of NLRP3, ASC and Caspase-1 in cells, and increased the secretion of inflammatory cytokines from cells. **A** Western blot analysis of NLRP3, ASC, and Caspase-1 in cells after treatment with different concentrations of UA (Full-length blots/gels are presented in Additional file 2: Fig. S2); **B** Quantification of NLRP3, ASC, and Caspase-1 in cells after treatment with different concentrations of UA; **C** Western blot analysis of NLRP3, ASC, and Caspase-1 in cells after incubation with UA for different times (Full-length blots/gels are presented in Additional file 3: Fig. S3); **D** Quantification of NLRP3, ASC, and Caspase-1 in cells after incubation with UA for different times; **E** Measurement of ICAM-1, MCP-1, and VCAM-1 in cells supernatant by ELISA after treatment with different concentrations of UA; **F** Measurement of ICAM-1, MCP-1, and VCAM-1 in cells supernatant after incubation with UA for different incubation time. *P < 0.05 and **P < 0.01 compared with group 0 mg/dL UA or group 0 h incubation

In view of the crucial role of NLRP3 inflammasome in modulation of inflammation, we assessed the status of NLRP3, Cacspase-1, and ASC followed treatment by different incubation time or different concentrations of UA (Fig. 2). We found that higher concentrations (8 and 12 mg/dL) of UA significantly increased the expression of ASC, Cacspase-1, and NLRP3 compared with lower concentrations (0 and 4 mg/dL) (Fig. 2A, B). Meanwhile, the protein expression of ASC increased significantly with the extension of UA incubation time at 12 mg/dL. However, the level of both ASC and Cacspase-1 increased remarkably only after longer UA incubation time (12 and 24 h) (Fig. 2C, D). Therefore, the stimulatory effects of UA on the expression of ASC, Cacspase-1, and NLRP3 is in time- and/or dose-dependent manners.

To determine whether UA treatment enhanced pro-inflammatory cytokine production, we analyzed various cytokines secretion from cells treated by UA (Fig. 2E, F) for 6 h. The content of MCP-1, ICAM-1 and VCAM-1 in supernatant remarkably increased in a dose-dependent manner (From 0 to 12 mg/dL) (Fig. 2E). We also observed that the content of MCP-1 and VCAM-1 increased markedly after 1 h incubation with 12 mg/dL UA. The concentration of ICAM-1 in supernatant increased gradually (Fig. 2F). Our data demonstrate that supplementation of UA augmented secretion of inflammatory cytokines.

### Knockdown of NLRP3 reversed the influence of UA on MIRI

**Fig. 3:**
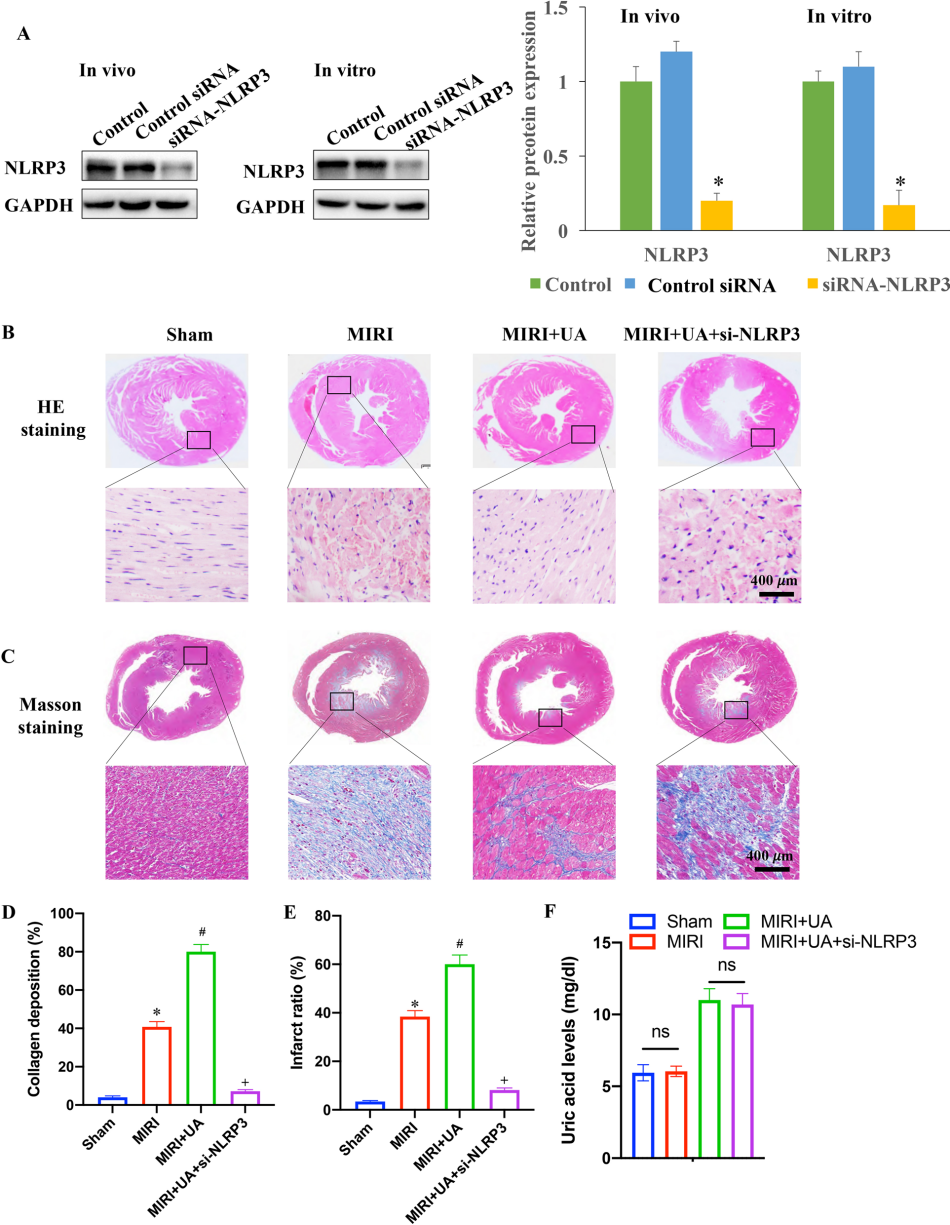
Knockdown of NLRP3 reversed the influence of UA on MIRI. **A** The knockdown of NLRP3 was validated in vivo and in vitro levels (Full-length blots/gels are presented in Additional file 4: Fig. S4); **B** HE staining was performed to investigate myocardial injury level; **C** Masson staining was performed to investigate collagen fibrotic area; **D** Collagen deposition was analyzed; **E** Infarct ratio was analyzed; **F** The serum level of UA was detected. *P < 0.05 compared with the group sham. #*P* < 0.05 compared with the group MIRI. + *P* < 0.05 compared with the group MIRI + UA. The animal numbers were 8 for each group (4 male rats and 4 female rats). Magnifications of HE and Masson staining are 10× or 400×

**Fig. 4:**
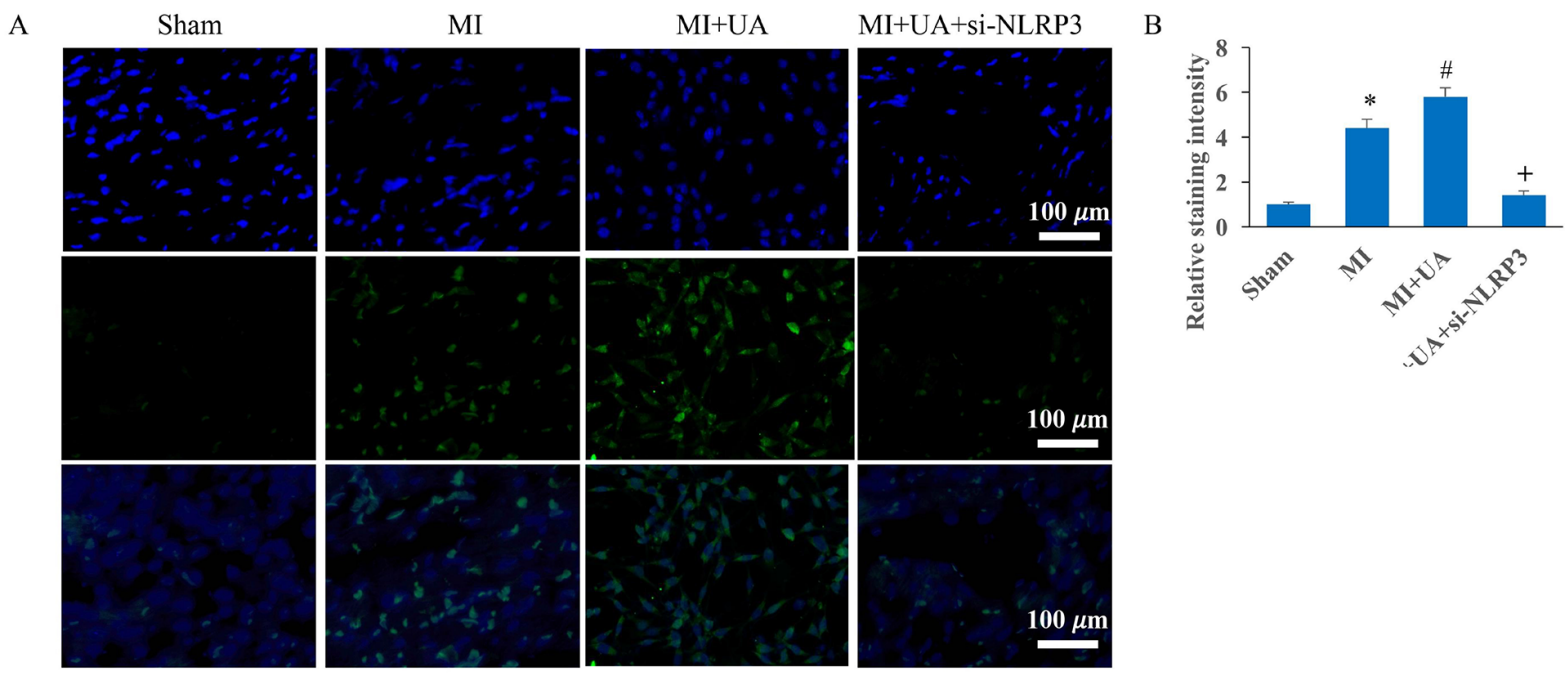
Knockdown of NLRP3 reversed the influence of UA on cardiomyocyte apoptosis. **A** Tunel staining was performed to investigate cardiomyocyte apoptosis; **B** Apoptosis intensity was analyzed. #*P* < 0.05 compared with the group MI. + *P* < 0.05 compared with the group MI + UA. The animal numbers were 8 for each group. Magnification of Tunel staining is 400×

Knockdown of NLRP3 vector was constructed with siRNA-NLRP3 (si-NLRP3), and the knockdown of NLRP3 was validated in vivo and in vitro levels (Fig. 3A). Disorderly arranged muscle fibers were observed in the MIRI group, and UA treatment aggravated these trend (Fig. 3B). However, si-NLRP3 treatment improve the myocardial tissue structure (Fig. 3B). Significant increase collagen deposition and fibrotic area (Fig. 3C–E) in the group MIRI were observed, and UA treatment remarkably promoted these damages. However, the levels of collagen deposition and fibrotic area were significantly decreased in the group MIRI + UA + is − NLRP3. Therefore, Knockdown of NLRP3 could improve the morphological damages induced by MIRI and UA. In addition, the level of UA in the serum was measured. The induction of MIRI didn’t promote the serum level of UA compared with group sham. Meanwhile, no significant difference was found between group MIRI + UA and group MIRI + UA + si − NLRP3 (Fig. 3F). The apoptosis intensity in the myocardial tissues was significantly promoted in the group MI, and aggravated after UA induction (Fig. 4A, B). However, si-NLRP3 remarkably reversed the influence of MIRI and UA on apoptosis, and suppressed apoptosis intensity (Fig. 4A, B).

### ***UA activated ROS/TRPM2/Ca***^***2***+^***pathway in AC16 and HAEC cell lines through targeting NLRP3***

**Fig. 5:**
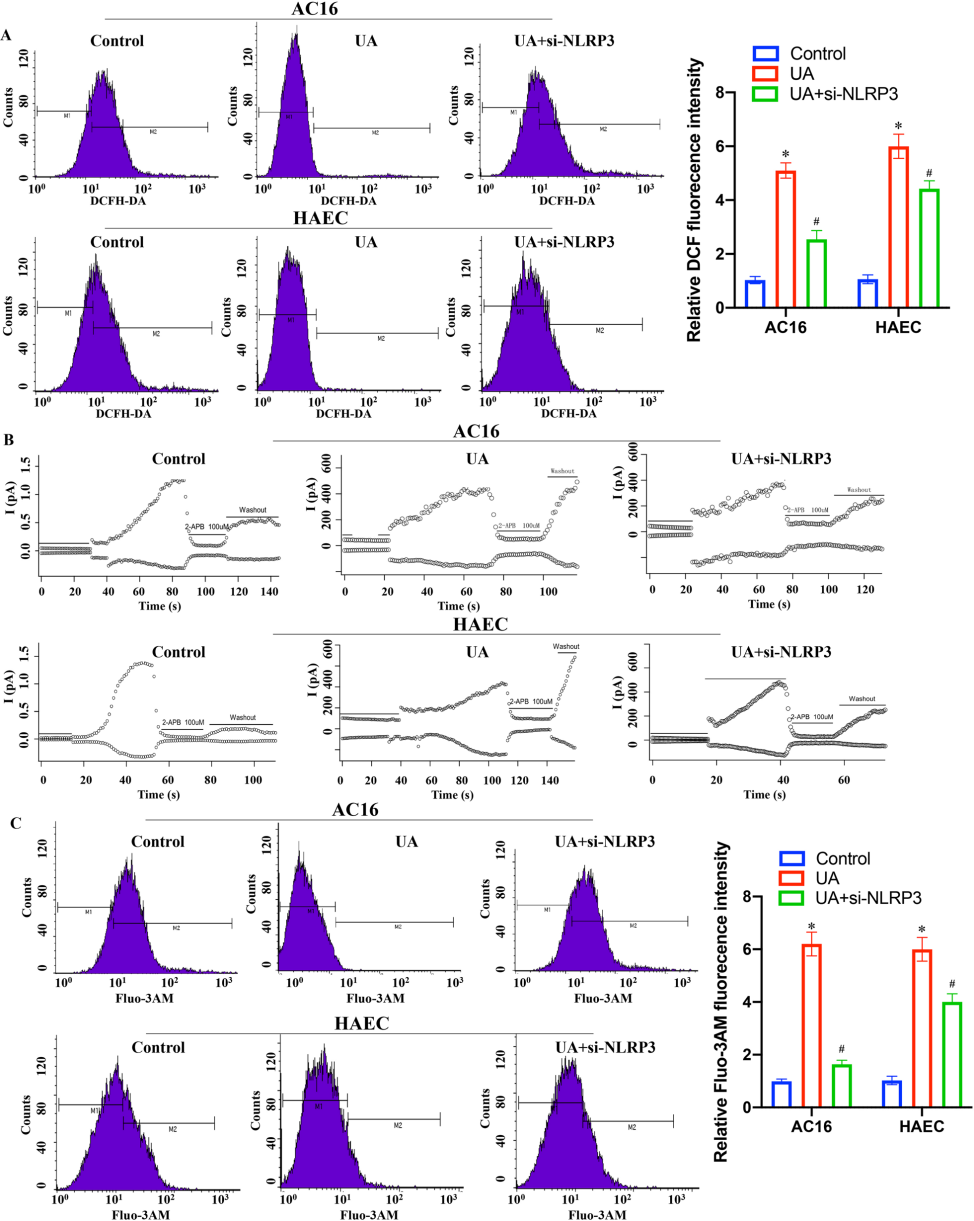
UA activated ROS/TRPM2/Ca^2+^ pathway in AC16 and HAEC cell lines through targeting NLRP3. **A** ROS production in mitochondrion was measured by flow cytometry; **B** Measurement of TRPM2 channel currents after different treatments; **C** Calcium ion influx in cells was measured by flow cytometry. *P < 0.05 compared with group control, ^#^*P* < 0.05 compared with group 12 mg/dL UA

It was reported that, high ROS production [29], TRPM2 channel currents [30], and calcium ion influx [31] could result in endothelial cell apoptosis and further accelerate MI. Therefore, the ROS production, TRPM2 channel currents, and calcium ion were measured after treatment with UA. High concentration of UA (12 mg/dL) markedly increased the ROS production, TRPM2 channel currents, and calcium ion influx in cells (Fig. 5). However, treatment with si-NLRP3 significantly reversed the influence of UA on ROS production (Fig. 5A), TRPM2 channel currents (Fig. 5B), and calcium ion influx (Fig. 5C). These findings indicate that UA may regulate ROS/TRPM2/Ca^2+^ pathway through targeting NLRP3.

### Knockdown of NLRP3 reversed the influence of UA on apoptosis and cell cycle in AC16 and HAEC cell lines

**Fig. 6:**
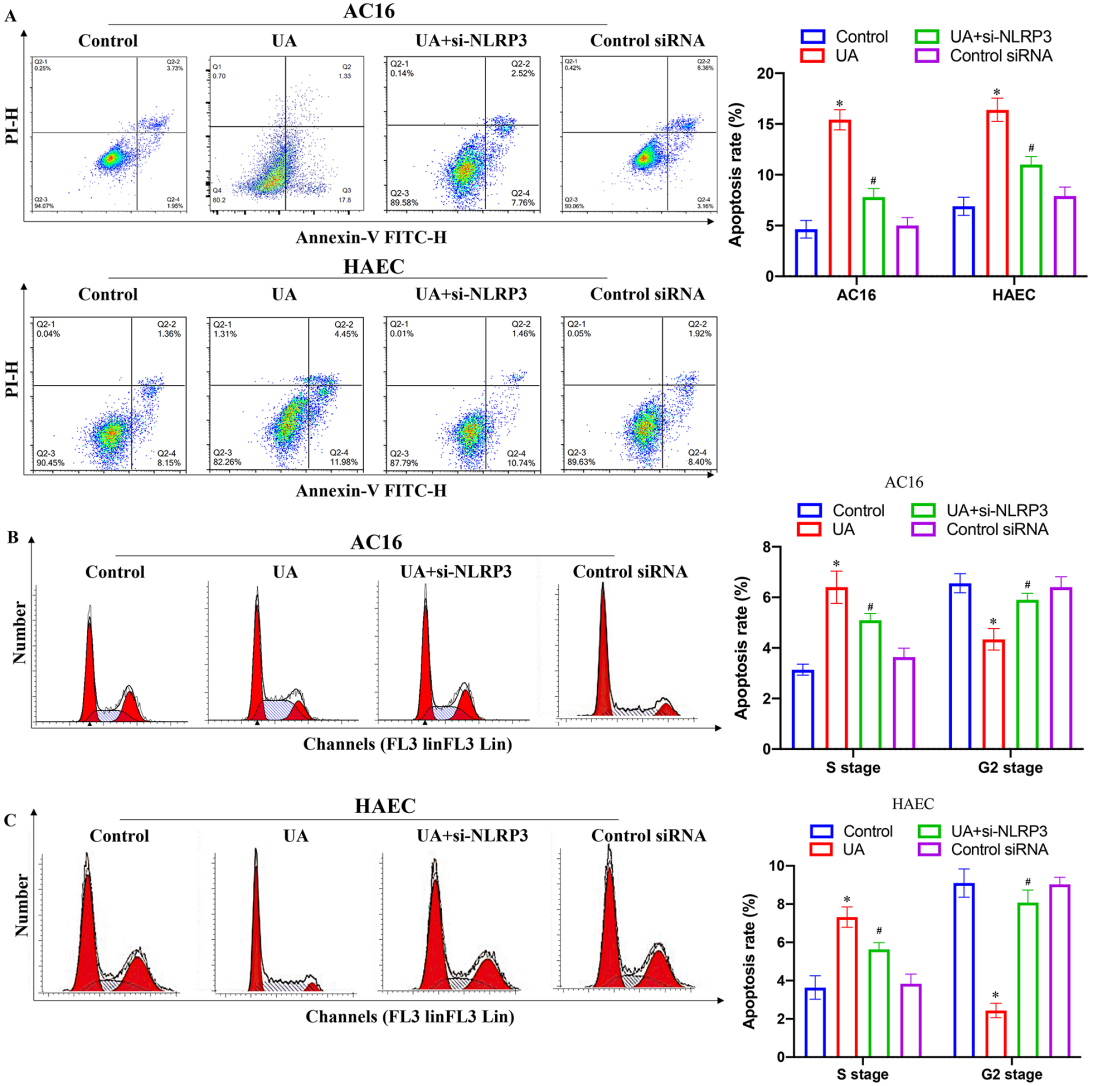
Knockdown of NLRP3 reversed the influence of UA on apoptosis and cell cycle in AC16 and HAEC cell lines. **A** Cell apoptosis was measured after different treatments; **B** Cell cycle of AC16 cell line was measured after different treatments; **C** Cell cycle of HAEC cell line was measured after different treatments. *P < 0.05 compared with the group control. #*P* < 0.05 compared with the group UA

The effects of si-NLRP3 on cell apoptosis and cell cycle in vitro were also investigated with both AC16 and HAEC cells. UA treatment significantly increased apoptosis rate and cell percentage in the S stage, but decreased cell percentage in the G2 stage compared to group control (Fig. 6A–C). However, si-NLRP3 markedly reversed the influence of UA with inhibiting cell apoptosis and cell percentage in the S stage, but increasing cell percentage in the G2 stage (Fig. 6A–C). Therefore, UA treatment might block the cells in S phase, but knockdown of NLRP3 could relieve the effect of UA.

## Discussion

It was reported that serum UA was positively related with the carotid intima-media thickness, which is considered to be associated with increasing risk of cardiovascular disease, and it is a sensitive marker of atherosclerosis [32, 33]. Meanwhile, UA could promote endothelial dysfunction, which is also a symbol pathological changes of atherosclerosis [34]. In this study, we proved that UA could promote apoptosis and inhibit proliferation of cells, which may account for its role in endothelial dysfunction.

Some inflammatory factors, such as ICAM-1, MCP-1, and VCAM-1, have the ability of promoting monocyte transmigration into the sub-endothelium and differentiation into macrophages [35, 36]. Then the macrophages take up oxidized low-density lipoprotein to become foam cells, the hallmark of atherosclerosis [37, 38]. Our findings demonstrated that high concentration of UA or longer incubation of it could promote the secretion of ICAM-1, MCP-1, and VCAM-1 from cells, and increase the expression of them in cells. We also identified this effect in vivo, and found that significant high expression of these chemokines and adhesion molecules in the carotid vascular walls could be induced by UA. Therefore, the promotion effects of UA on the secretion and expression of inflammatory factors may also explain its role in the atherosclerosis. In this study, we found that UA indeed damaged cardiomyocytes, and increased the levels of some inflammatory factors including MCP-1, VCAM-1, and ICAM-1. However, if UA directly damaged cardiomyocytes, or the increased inflammatory condition leaded to worse atherosclerosis, or perhaps MI-induced inflammation leaded to worse outcomes remain unclear. We are inclined to this view that UA synergizes with increased inflammatory condition caused by UA and MI injury to result in worse outcomes, which need to be further investigated.

Previous report proved that high NLRP3 inflammasome expression in the aorta was identified in the patients with coronary atherosclerosis, and the NLRP3 expression was correlated with the severity of coronary artery stenosis [39, 40]. In this study, we demonstrated that high expression of NLRP3, ASC, and Caspase 1 in cells and carotid vascular wall could be induced by UA. Therefore, UA might influence the development and progression of atherosclerosis through activating NLRP3 inflammasome.

Previous study indicated that TRPM2 channel/Ca^2+^ influx influenced the ROS-induced signaling cascade responsible for chemokine production, which promoted inflammation [17]. Meanwhile, Ca^2+^ influx through TRPM2 channel has been considered to be closely linked with cell death and neurodegenerative diseases [41], and the activation of TRPM2 channel is regulated by oxidative stress [16]. We firstly demonstrated that ROS/TRPM2 channel/Ca^2+^ pathway could be modulated by high concentration of UA through targeting NLRP3. Therefore, the cardiomyocytes injury caused by UA may be induced by the activation of NLRP3 and ROS/TRPM2 channel/Ca^2+^. The in vitro studies were performed in human cardiomyocyte cell line (AC16) and not in rat adult cardiomyocytes, which is a major limitation. In addition, the apoptosis and necrosis seen in MIRI might be caused by ischemic injury resulting from thrombosis of the atherosclerotic coronary vessel, which needs to be further studied.

Chronic kidney disease is a known atherosclerotic risk factor, and also leads to hyperuricemia. It was reported that UA levels were significantly higher in patients with acute myocardial infarction, and hyperuricemia was closely associated with acute myocardial infarction [42]. In addition, hyperuricemia is significantly associated with hypertension, renal dysfunction and independently confers a higher risk of mortality in patients with acute myocardial infarction [43]. Whether patients with hyperuricemia alone would also have a bad impact on their post-MI outcomes needs to be further investigated.

## Conclusion

The findings of the present study show that UA treatment activates NLRP3 inflammasome, promotes cell death, and increases the production of inflammatory factors, thereby leading to MI. Additionally, UA may contribute to cardiomyocytes injury through activating NLRP3 inflammasome and ROS/TRPM2/Ca^2+^ pathway. These findings provide specific evidences about how UA influences the cardiomyocytes injury. Therefore, the control of serum UA should be emphasized and intervened in patients with MI risk. In the primary care setting, patients with MI risk should be tested for hyperuricemia and treated to prevent worse outcomes. This research provides a novel thought for the therapeutic strategy of MI injury by regulating NLRP3/ROS/TRPM2/Ca^2+^.

## Supplementary Information

**Additional file 1. Supplementary Figure 1.** Identification of cardiomyocytes with connexin 43 and SIRP-*α* antibodies.

**Additional file 2. Supplementary Figure 2.** Full-length blots/gels of Figure 2 A.

**Additional file 3. Supplementary Figure 3.** Full-length blots/gels of Figure 2 C.

**Additional file 4. Supplementary Figure 4.** Full-length blots/gels of Figure 3 A.

**Additional file 5. Supplementary Figure 5.** ST elevation electrocardiogram data.

## Data Availability

The datasets used and/or analysed during the current study are available from the corresponding author on reasonable request.

## Abbreviations

MI: Myocardial infarction
UA: Uric acid
NLRP3: Pyrin domain-containing 3
ASC: Apoptosis­associated speck­like protein
MCP-1: Monocyte chemoattractant protein-1
ICAM-1: Intercellular adhesion molecule-1
VCAM-1: Vascular cell adhesion molecule-1
si-NLRP3: SiRNA-NLRP3
