# Correction: Uric acid promotes myocardial infarction injury via activating Pyrin domain-containing 3 inflammasome and reactive oxygen species/transient receptor potential melastatin 2/Ca2 + pathway

**DOI:** 10.1186/s12872-026-05613-2

**Published:** 2026-04-11

**Authors:** Haiyun Wu, Ruozhu Dai, Min Wang, Chengbo Chen

**Affiliations:** Department of Cardiology, Quanzhou First Hospital Affiliated to Fujian Medical University, No. 250 East Street, Quanzhou, 362000 China

BMC Cardiovascular Disorders (2023) 23:10

10.1186/s12872-023-03040-1

In this article [1], the image of group 0 h in the figure 1B, the image of group MI+UA in the Figure 4A, and the image of group UA in the figure 6A (AC16 cell) were appeared incorrectly and have now been corrected in the original publication. For completeness and transparency, the old incorrect versions are displayed below.

The original article has been corrected.

Incorrect Figures:

**Fig. 1:**
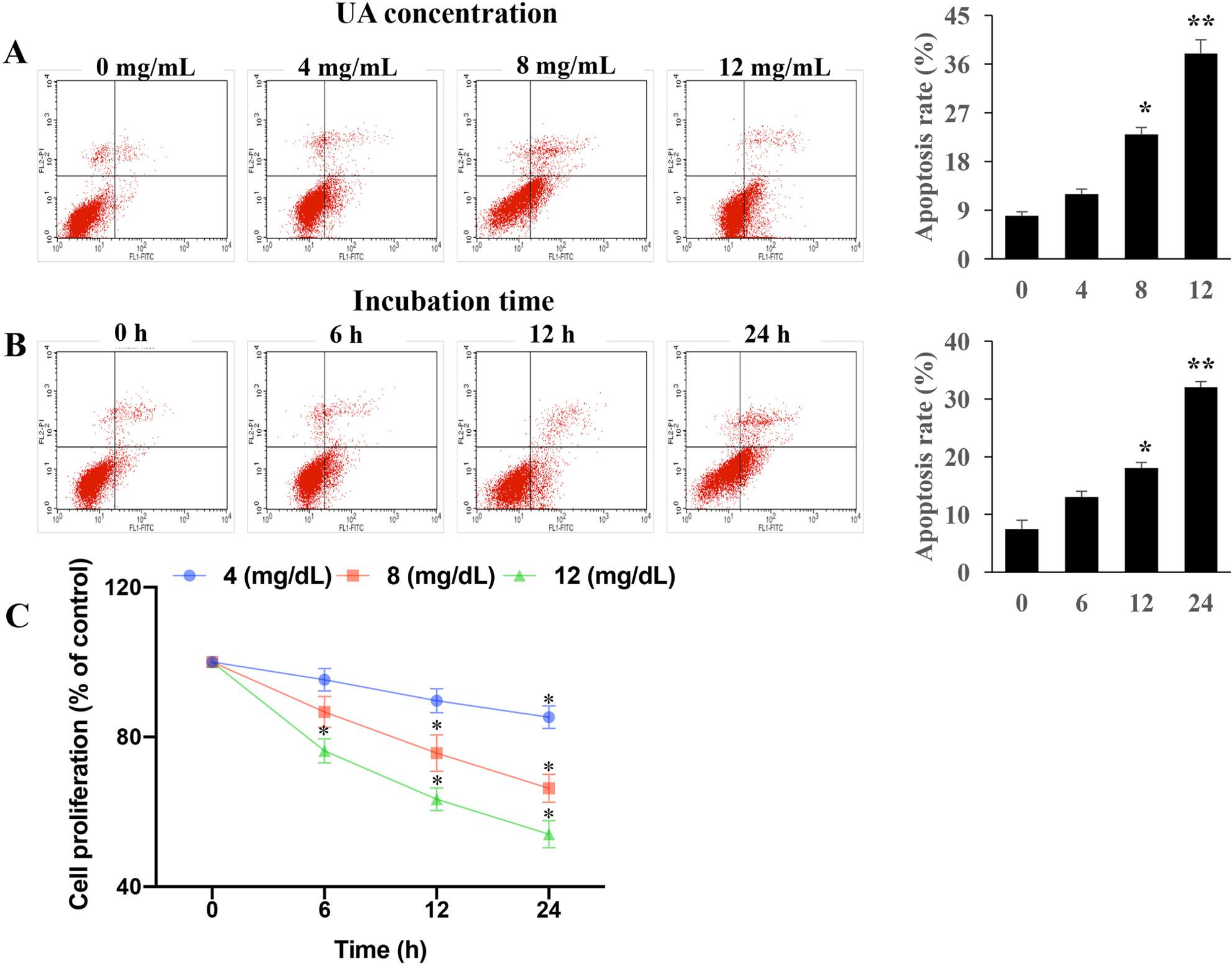
UA promoted apoptosis and inhibited cell proliferation. **A** UA enhanced the apoptosis of cells through dose-dependent manner; **B** UA promoted the apoptosis of cells through time-dependent manner; **C** UA suppressed the proliferation of cells. *P < 0.05 and **P < 0.01 compared with group 0 mg/dL UA or group 0 h incubation

**Fig. 4:**
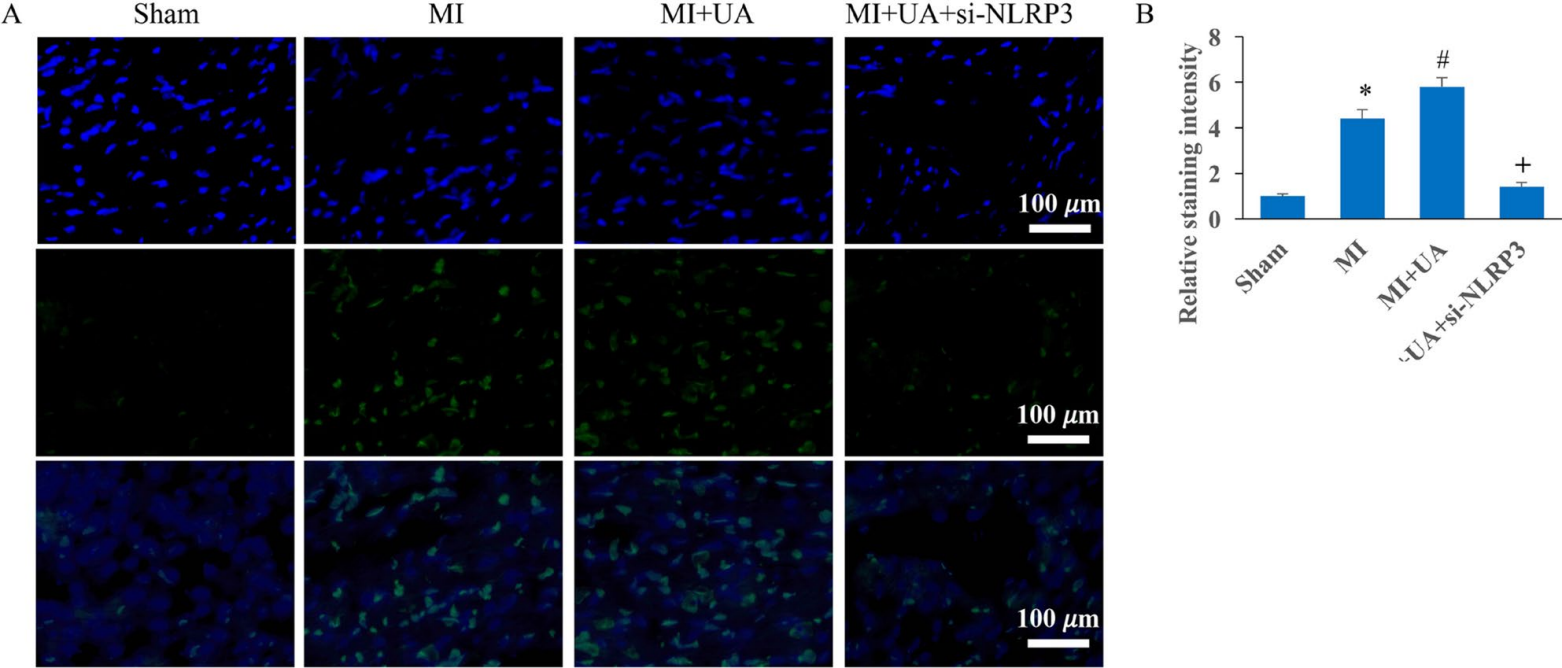
Knockdown of NLRP3 reversed the influence of UA on cardiomyocyte apoptosis. **A** Tunel staining was performed to investigate cardiomyocyte apoptosis; **B** Apoptosis intensity was analyzed. #*P* < 0.05 compared with the group MI. + *P* < 0.05 compared with the group MI + UA. The animal numbers were 8 for each group. Magnification of Tunel staining is 400×

**Fig. 6:**
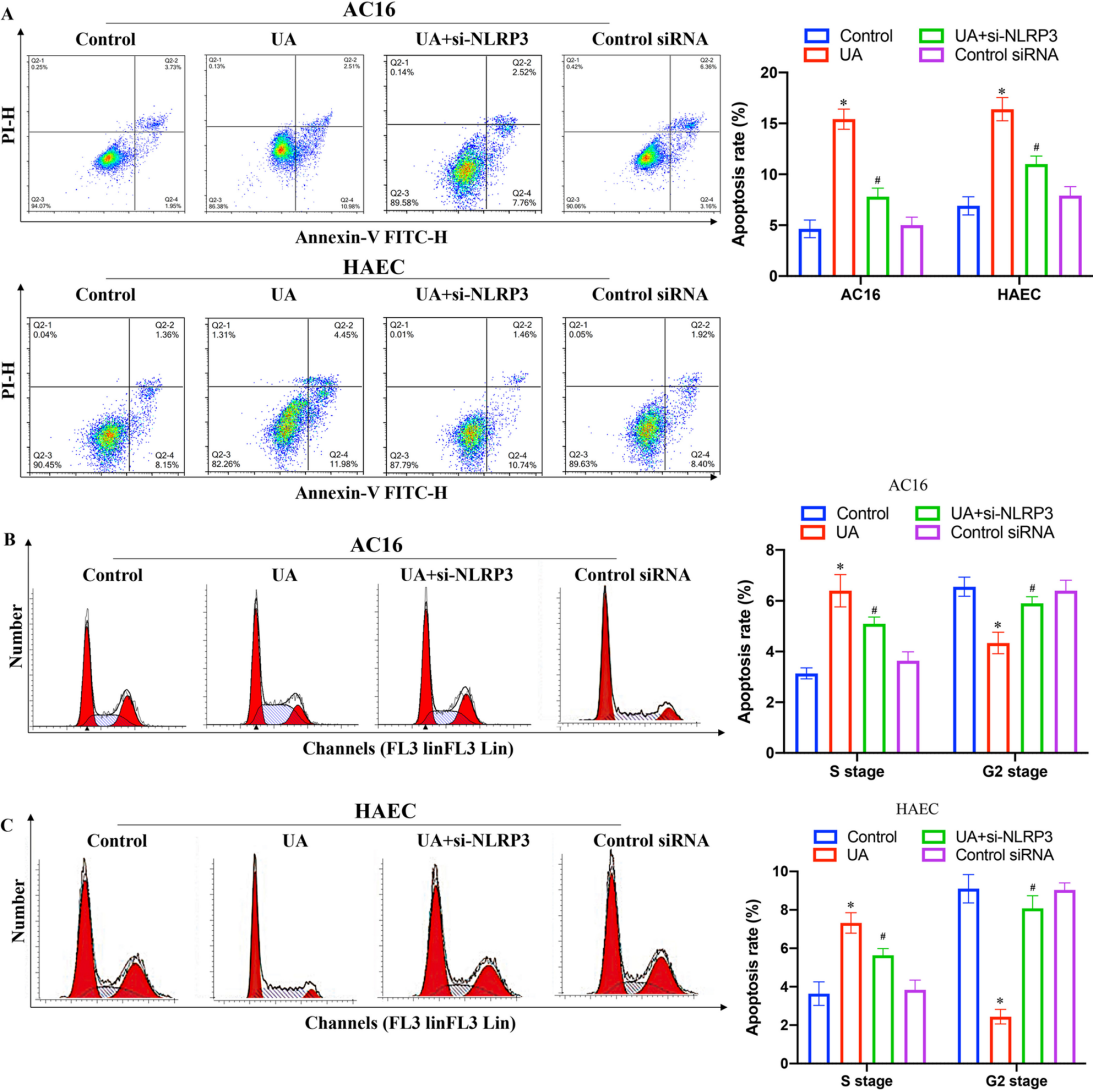
Knockdown of NLRP3 reversed the influence of UA on apoptosis and cell cycle in AC16 and HAEC cell lines. **A** Cell apoptosis was measured after different treatments; **B** Cell cycle of AC16 cell line was measured after different treatments; C Cell cycle of HAEC cell line was measured after different treatments. *P < 0.05 compared with the group control. #*P* < 0.05 compared with the group UA

Corrected Figures:

**Fig.1:**
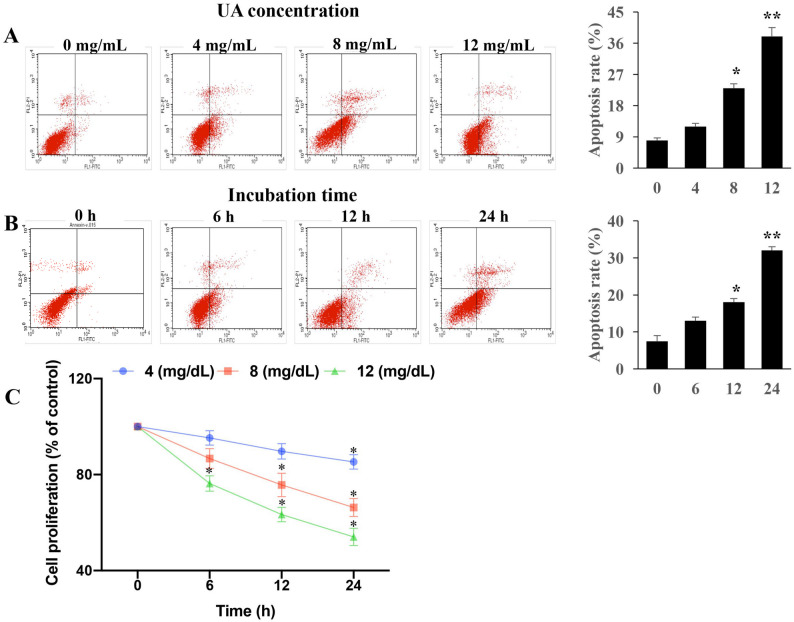
UA promoted apoptosis and inhibited cell proliferation. **A** UA enhanced the apoptosis of cells through dose-dependent manner; **B** UA promoted the apoptosis of cells through time-dependent manner; **C** UA suppressed the proliferation of cells. *P < 0.05 and **P < 0.01 compared with group 0 mg/dL UA or group 0 h incubation

**Fig. 4:**
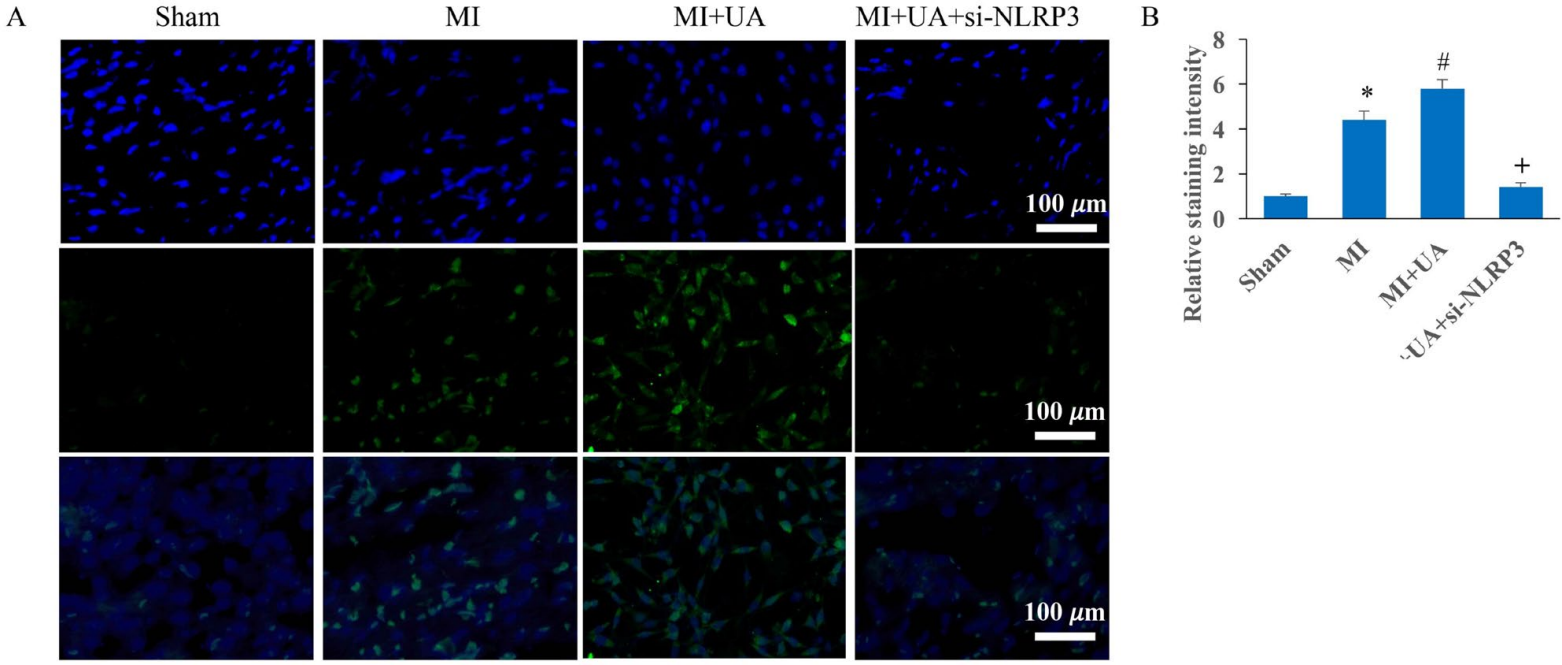
Knockdown of NLRP3 reversed the influence of UA on cardiomyocyte apoptosis. **A** Tunel staining was performed to investigate cardiomyocyte apoptosis; **B** Apoptosis intensity was analyzed. #*P* < 0.05 compared with the group MI. + *P* < 0.05 compared with the group MI + UA. The animal numbers were 8 for each group. Magnification of Tunel staining is 400×

**Fig. 6:**
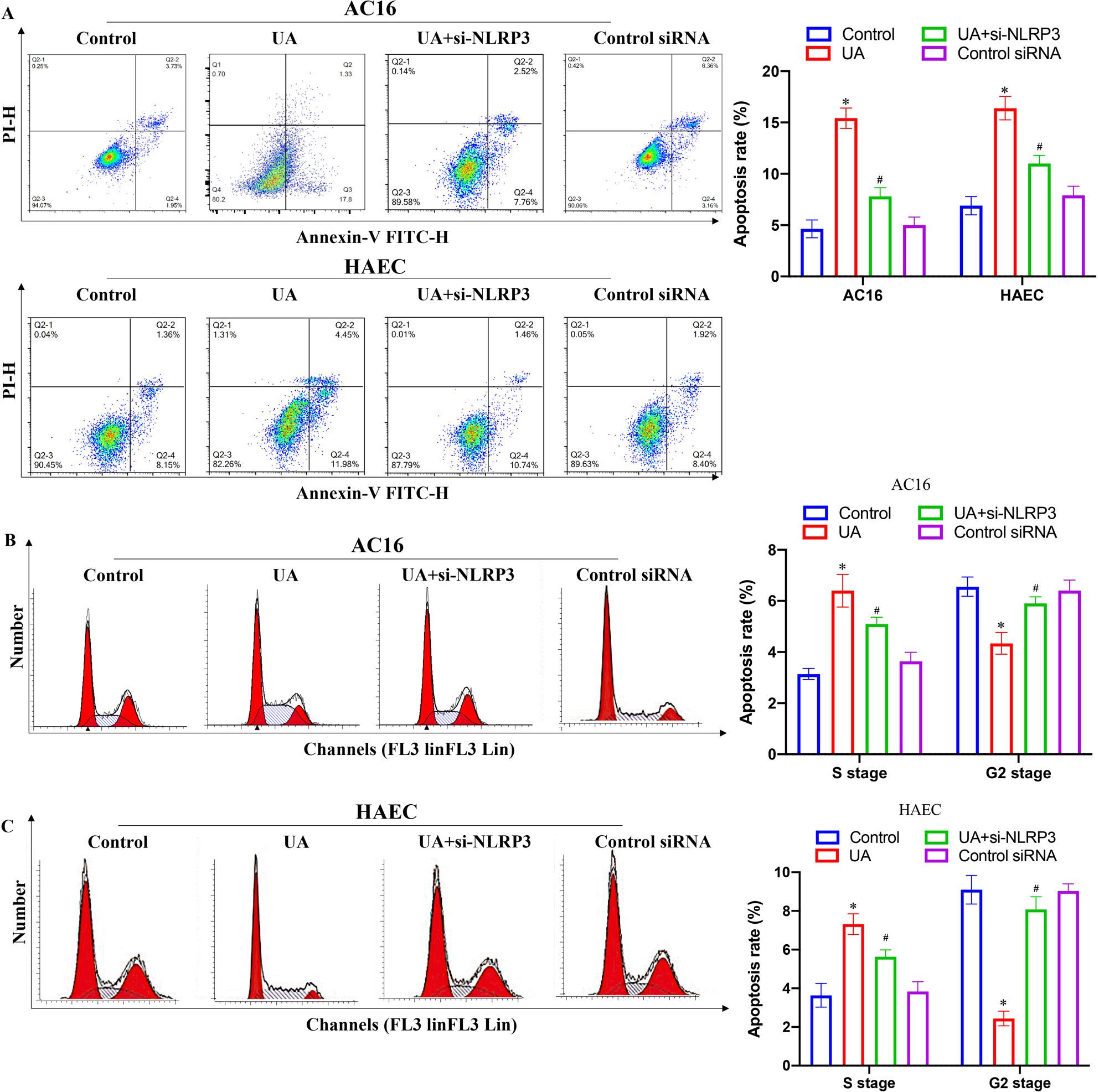
Knockdown of NLRP3 reversed the influence of UA on apoptosis and cell cycle in AC16 and HAEC cell lines. **A** Cell apoptosis was measured after different treatments; **B** Cell cycle of AC16 cell line was measured after different treatments; **C** Cell cycle of HAEC cell line was measured after different treatments. *P < 0.05 compared with the group control. #*P* < 0.05 compared with the group UA
