# Supplementary figures and images for: Uric acid promotes myocardial infarction injury via activating pyrin domain-containing 3 inflammasome and reactive oxygen species/transient receptor potential melastatin 2/Ca2+pathway
Source: BMC Cardiovasc Disord. 2023 Jan 10;23:10. doi: 10.1186/s12872-023-03040-1 (PMC9830724; doi:10.1186/s12872-023-03040-1)

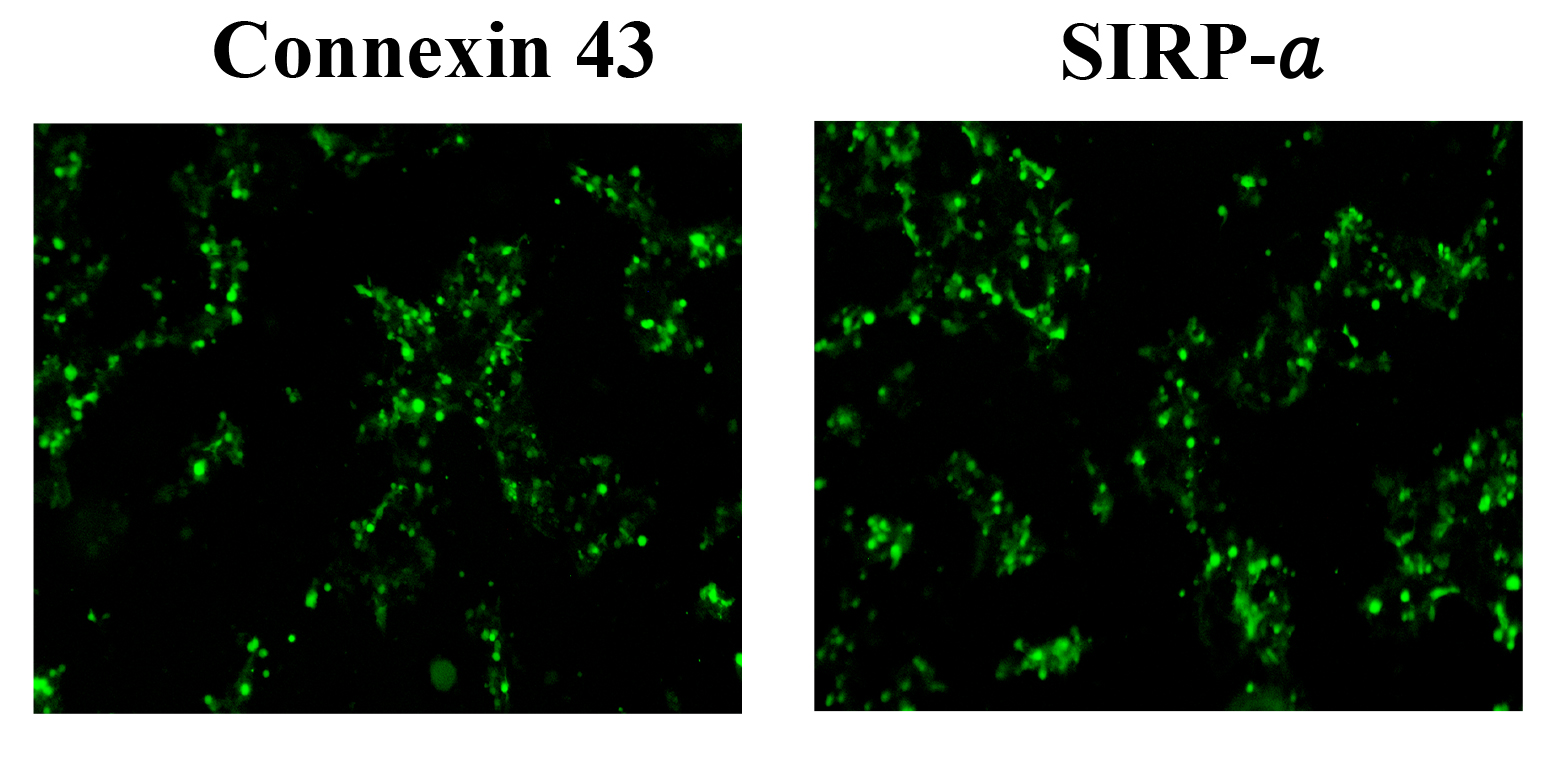

Supplement: Supplementary file 1 — Additional file 1. Supplementary Figure 1. Identification of cardiomyocytes with connexin 43 and SIRP-α antibodies. [file 12872_2023_3040_MOESM1_ESM.jpg]

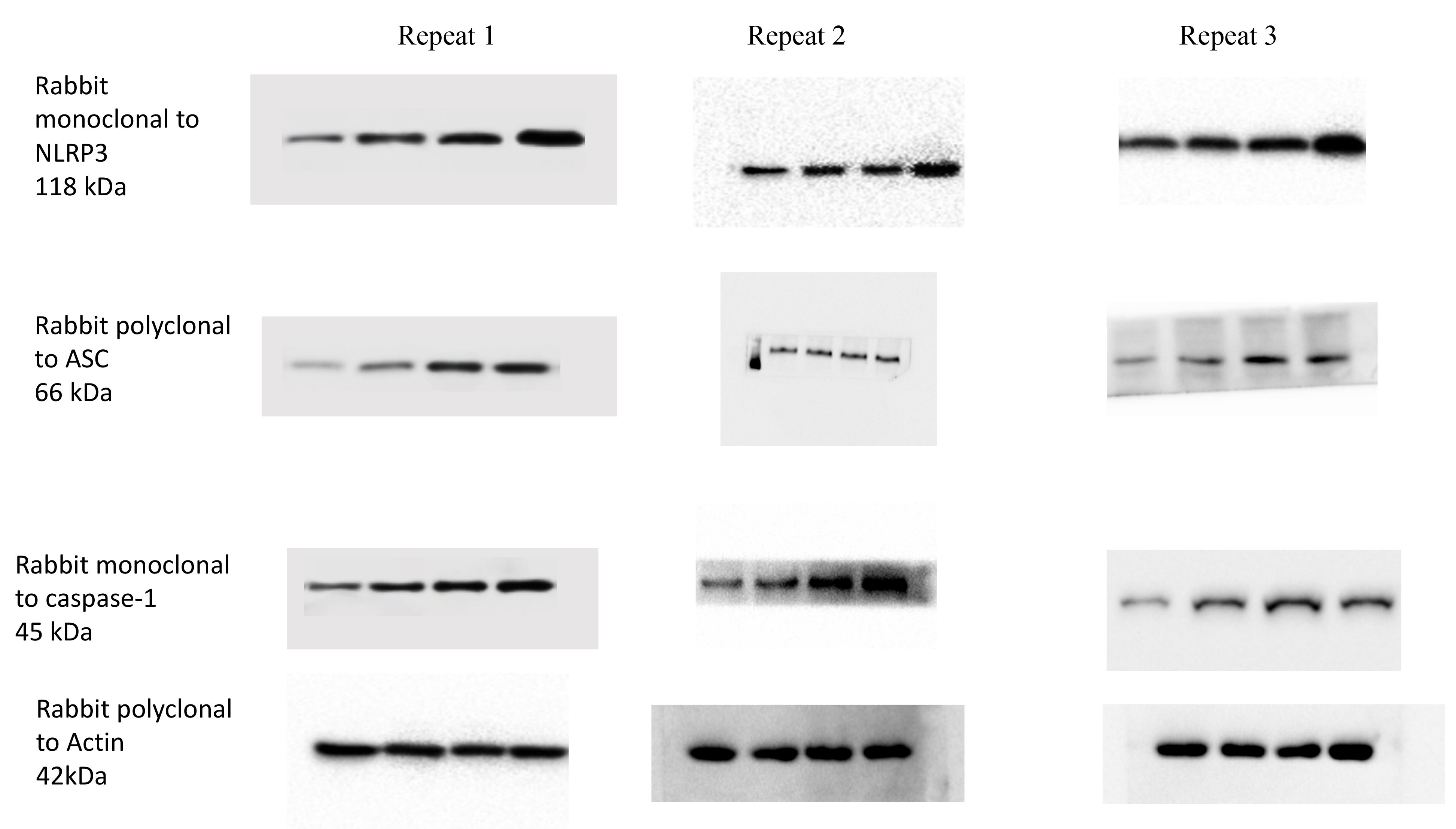

Supplement: Supplementary file 2 — Additional file 2. Supplementary Figure 2. Full-length blots/gels of Figure 2 A. [file 12872_2023_3040_MOESM2_ESM.jpg]

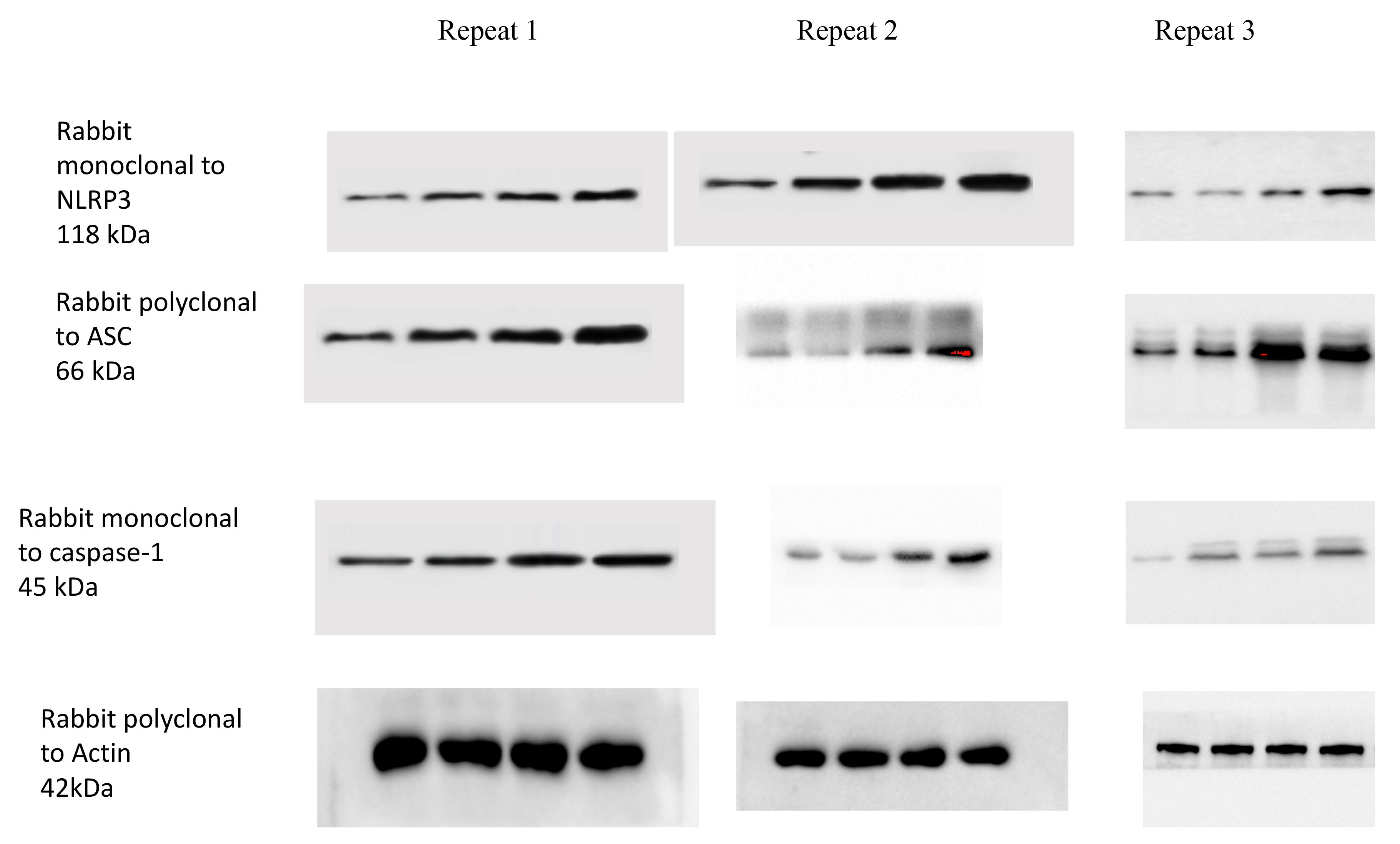

Supplement: Supplementary file 3 — Additional file 3. Supplementary Figure 3. Full-length blots/gels of Figure 2 C. [file 12872_2023_3040_MOESM3_ESM.jpg]

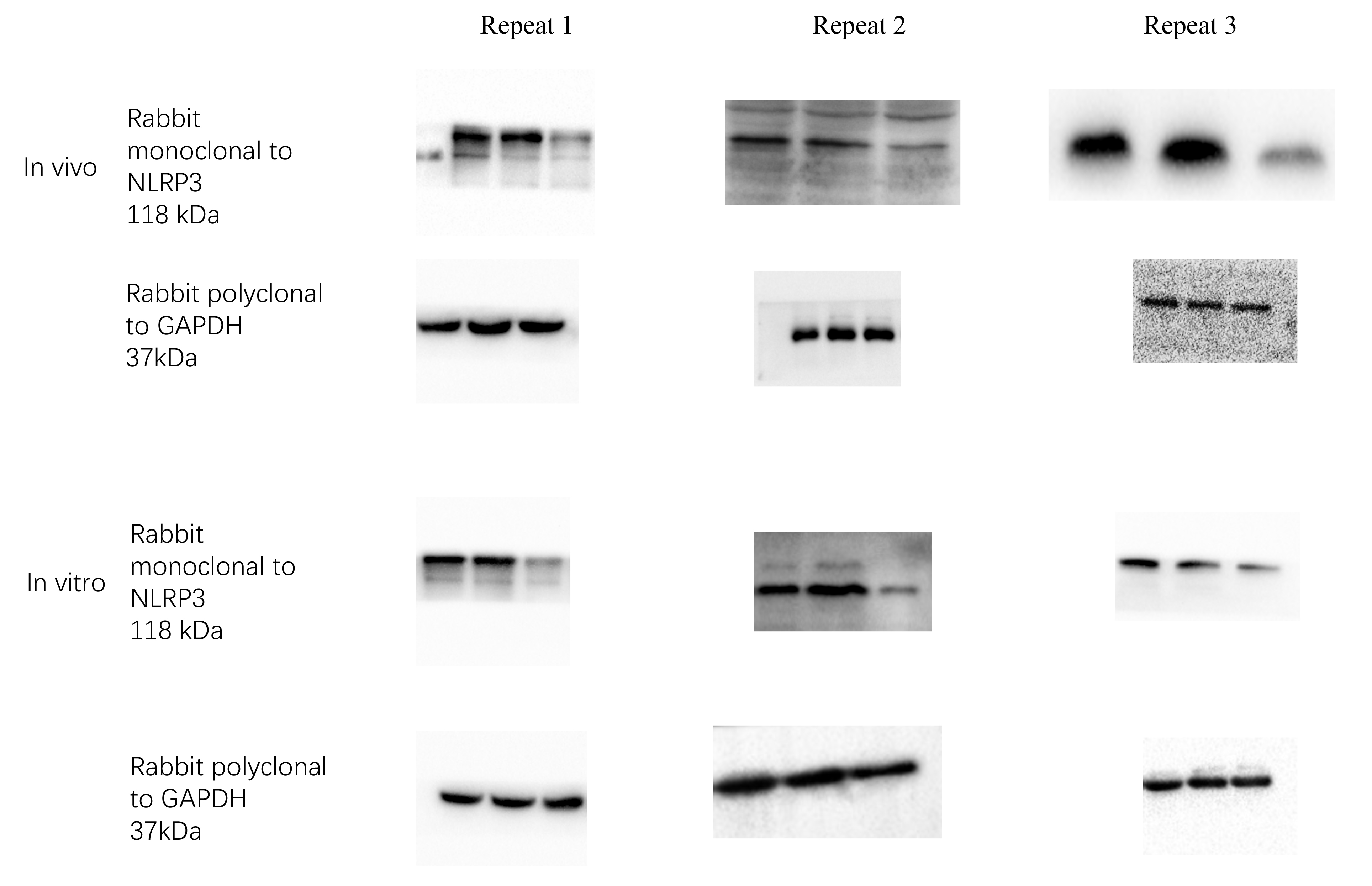

Supplement: Supplementary file 4 — Additional file 4. Supplementary Figure 4. Full-length blots/gels of Figure 3 A. [file 12872_2023_3040_MOESM4_ESM.jpg]

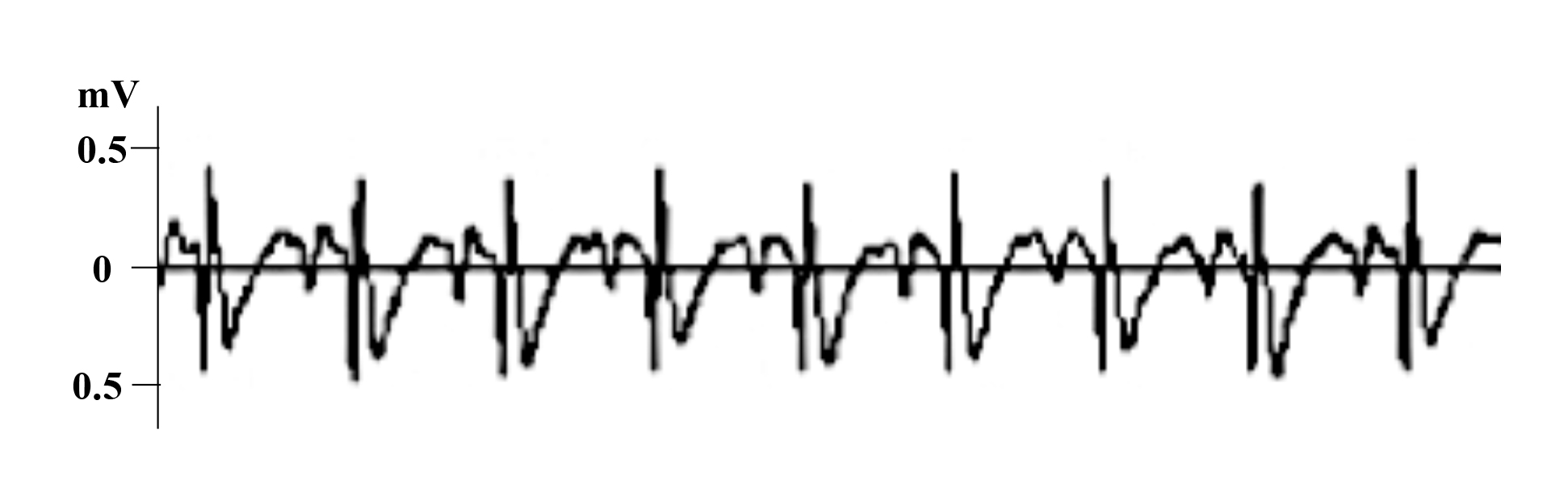

Supplement: Supplementary file 5 — Additional file 5. Supplementary Figure 5. ST elevation electrocardiogram data. [file 12872_2023_3040_MOESM5_ESM.jpg]
